# Impact of residual and intrafractional errors on strategy of correction for image-guided accelerated partial breast irradiation

**DOI:** 10.1186/1748-717X-5-96

**Published:** 2010-10-26

**Authors:** Gang Cai, Wei-Gang Hu, Jia-Yi Chen, Xiao-Li Yu, Zi-Qiang Pan, Zhao-Zhi Yang, Xiao-Mao Guo, Zhi-Min Shao, Guo-Liang Jiang

**Affiliations:** 1 Department of Radiation Oncology, Cancer Hospital, Department of Oncology, Shanghai Medical college, Fudan University, Shanghai, China; 2 Department of Breast Surgery, Cancer Hospital, Department of Oncology, Shanghai Medical college, Fudan University, Shanghai, China

## Abstract

**Background:**

The cone beam CT (CBCT) guided radiation can reduce the systematic and random setup errors as compared to the skin-mark setup. However, the residual and intrafractional (RAIF) errors are still unknown. The purpose of this paper is to investigate the magnitude of RAIF errors and correction action levels needed in cone beam computed tomography (CBCT) guided accelerated partial breast irradiation (APBI).

**Methods:**

Ten patients were enrolled in the prospective study of CBCT guided APBI. The postoperative tumor bed was irradiated with 38.5 Gy in 10 fractions over 5 days. Two cone-beam CT data sets were obtained with one before and one after the treatment delivery. The CBCT images were registered online to the planning CT images using the automatic algorithm followed by a fine manual adjustment. An action level of 3 mm, meaning that corrections were performed for translations exceeding 3 mm, was implemented in clinical treatments. Based on the acquired data, different correction action levels were simulated, and random RAIF errors, systematic RAIF errors and related margins before and after the treatments were determined for varying correction action levels.

**Results:**

A total of 75 pairs of CBCT data sets were analyzed. The systematic and random setup errors based on skin-mark setup prior to treatment delivery were 2.1 mm and 1.8 mm in the lateral (LR), 3.1 mm and 2.3 mm in the superior-inferior (SI), and 2.3 mm and 2.0 mm in the anterior-posterior (AP) directions. With the 3 mm correction action level, the systematic and random RAIF errors were 2.5 mm and 2.3 mm in the LR direction, 2.3 mm and 2.3 mm in the SI direction, and 2.3 mm and 2.2 mm in the AP direction after treatments delivery. Accordingly, the margins for correction action levels of 3 mm, 4 mm, 5 mm, 6 mm and no correction were 7.9 mm, 8.0 mm, 8.0 mm, 7.9 mm and 8.0 mm in the LR direction; 6.4 mm, 7.1 mm, 7.9 mm, 9.2 mm and 10.5 mm in the SI direction; 7.6 mm, 7.9 mm, 9.4 mm, 10.1 mm and 12.7 mm in the AP direction, respectively.

**Conclusions:**

Residual and intrafractional errors can significantly affect the accuracy of image-guided APBI with nonplanar 3DCRT techniques. If a 10-mm CTV-PTV margin is applied, a correction action level of 5 mm or less is necessary so as to maintain the RAIF errors within 10 mm for more than 95% of fractions. Pre-treatment CBCT guidance is not a guarantee for safe delivery of the treatment despite its known benefits of reducing the initial setup errors. A patient position verification and correction during the treatment may be a method for the safe delivery.

## Background

Several groups have shown that accelerated partial breast irradiation (APBI) for selected patients have comparable outcome to the standard whole breast irradiation after breast conservative surgery [1-3]. The three-dimensional conformal radiotherapy (3DCRT) has shown the advantages of noninvasive and easy implementation in a modern radiotherapy department [4,5]. According to the RTOG 0319 report [6], APBI has achieved similar early outcomes as whole-breast irradiation (WBI). Various techniques have been adopted in the 3DCRT, including the multiple noncoplanar field technique, three-field mixed modality technique and proton therapy [7-12].

Compared to WBI, APBI requires more accuracy because the highly conformal dose is delivered to a relatively small area. The cone beam CT (CBCT) guided radiation therapy has been used to reduce the probability of geographical displacements in different sites[13-19]. White et al. reported that CBCT guided setup with an action level of 3 mm could reduce the systematic and random setup errors as compared to the skin-mark setup[20], but no data on intra-fractional error was reported. Also, some errors would still exist after the couch shift, which are named as residual errors, therefore, it is unclear whether the magnitude of residual and intrafractional errors would significantly affect the correction levels and the appropriate planning target margins. This is especially necessary for APBI treated with noncoplanar fields. We therefore investigated the magnitude of residual and intrafractional errors with various pre-treatment correction action levels so as to determine the appropriate margins needed for CBCT guided APBI with noncoplanar 3DCRT techniques.

## Methods

### Patient eligibility

From July 2008 to December 2008, ten patients were enrolled in a prospective, single institutional review board-approved trial of APBI with CBCT imaging guidance. The eligibility criteria included: age ≥45 years, Stage T1N0M0 or Stage Tis, negative surgical margins (≥2 mm) after definitive surgery, and at least 4 titanium clips were placed in the resection cavity. The median age of the 10 enrolled patients was 55 (45-75). Four patients were diagnosed with ductal carcinoma in situ (DCIS), and the remaining 6 were diagnosed with invasive ductal carcinoma. One patient had the tumor on the right side, and the other 9 had their tumors on the left-side. Three patients (including one with DCIS) underwent sentinel lymph nodes biopsy, 4 patients with invasive carcinoma had axillary nodes dissection, and the other 3 DCIS had no axillary surgery. The primary tumor was located in the upper-outer quadrant in 3 patients, in the inner-upper quadrant in 4 patients and in the central or aerolar region in other 3 patients.

### Target delineation and treatment planning

All patients were immobilized using a Med-Tec 350 breastboard (Med-Tec Corporation, Orange, IA, USA) with both arms raised above their heads. CT images were acquired with 5-mm-thick intervals from the level of mandible through the lung base using a Philips big core CT scanner (Philips Medical Madison, Fitchburg, WI, USA). All CT images were exported to the Pinnacle treatment planning system (Philips Radiation Oncology Systems, Pinnacle version 8.0, Milpitas, CA) for contouring and treatment planning.

The lumpectomy/surgical cavity, ipsilateral breast, contralateral breast, lungs, heart, clinical target volume (CTV) and planning target volume (PTV) were segmented in the CT images. The CTV was the surgical cavity defined by clips and seroma plus a margin of 10 mm. An additional margin of 10 mm was placed around CTV to define PTV. Both CTV and PTV were limited to 5 mm from the skin surface and 5 mm from the lung-chest interface following the RTOG 0319 guideline. The ipsilateral and contralateral breasts were contoured with all the visible breast tissue on CT images, which extends from the infra-mammary fold to the head of clavicle in the cranial-caudal direction. The heart was contoured from the first CT slice below the pulmonary artery to the apex inferiorly. Both lungs were contoured in their entirety.

A 3DCRT technique using 6MV photons with 5-field non-coplanar beam arrangement was developed. The arrangement used fields that approximate breast tangents with a 15-20 degree steeper gantry angle for medial beams and couch angles of 15-70 degrees, similar to the report of Baglan et al.[12]. The treatment plans were manually optimized such that more than 95% of PTV was completely encompassed by the 95% isodose line, while maintaining a minimum dose greater than 93% and a maximum dose less than 110%. The dose prescription was 38.5 Gy delivered in 10 fractions, with a total duration of 5-7 days. The treatment was delivered twice daily with an interfractional interval of at least 6 hours.

The tolerances of normal tissues were defined as follows: 1) less than 10% of the ipsilateral lung receiving 30% of the prescribed dose (V-10% < 30%), 2) less than 10% of the contralateral lung receiving 5% of the prescribed dose, 3) less than 5% of the heart receiving 5% of the prescribed dose for right-sided patients, and 4) the volume of the heart receiving 5% of the prescribed dose should be below that for whole breast irradiation for patients with left-sided tumors.

All treatments were delivered with an Elekta Synergy S linear accelerator equipped with an electronic portal imaging device (EPID) and a kilovoltage cone-beam CT system (Elekta Synergy S, Elekta Oncology Systems, Crawley, UK). Three skin markers corresponding to the laser in the treatment room were used for initial setup.

### kVCBCT images acquisition and registration

Two kVCBCT imaging protocols were created separately for the left and right breast tumors. Both protocols had the parameters of F0 filter, S20 collimator, 120 kV, 36.1 mA-s and Med_Res reconstruction. The acquisition angles range from 250° to 90° (clockwise) for the left breast tumors and from 180° to 30° (clockwise) for the right breast tumors.

The first CBCT images were acquired immediately after positioning the patients with 3 skin markers. The CBCT images were first automatically registered to the planning CT using the grey value algorithm implemented in the XVI software (XVI, version 3.5 b147) followed by a manual fine adjustment to get a better match on chest wall, clips and skin in the axial, coronal and sagittal planes. All the online registration was done within 2-3 minutes by the same radiation oncologist. The isocenter was used as the correction reference point and all the rotational errors were disregarded. A couch shift was applied if the required shift was greater than 3 mm in any of the three directions. This threshold was designated as the 3 mm correction action level (3 mmCAL). Two therapists shifted the couch to the required position indicated by the XVI software, and the couch position was double checked by the radiation oncologist. A post-treatment CBCT with the same parameters was acquired after the treatment delivery. The same radiation oncologist performed the identical registration process and recorded the results.

### Correction action levels

The residual and intrafraction (RAIF) errors with the 3 mmCAL can be obtained directly from the first and second CBCT images. In addition, we simulated the hypothetical RAIF errors with increasing correction action levels(CAL) at 4 mm CAL, 5 mmCAL, 6 mmCAL and no correction (skin markers only). The process applied was:

(1) Register the first CBCT images (named CBCT1) with planning CT images and record the shifts in the lateral (LR), superior-inferior (SI) and anterior-posterior (AP) directions.

(2) Calculate and simulate the residual errors with different action levels after couch shift. In the 3 mmCAL (as with 4 mm, 5 mm and 6 mmCAL), any required shifts larger than 3 mm (4 mm, 5 mm and 6 mm) in any of the three directions will be set to zero and then saved in a new data set named 3 mmresidual (4 mmresidual, 5 mmresidual and 6 mmresidual). For example, if the results from the first registration were 3.5, 5 and 2 mm in the LR, SI and AP directions respectively, the 3 mmresidual would be 0,0 and 2 mm and the 4 mmresidual would be 3.5,0 and 2 mm.

(3) Register the post-treatment CBCT images (CBCT2) with planning CT and record the second shifted dataset in the LR, SI and AP directions.

(4) Calculate the intrafractional error (named Intraerror) using CBCT2 minus 3 mmresidual. In principle, the intrafractional error is independent of the correction levels.

(5) The residual and intrafractional (RAIF) errors in 3 mmCAL and other hypothetical correction action levels (4 mm, 5 mm, 6 mmCAL and nocorrection) were calculated by summing up the 4 mmresidual, 5 mmresidual, 6 mmrediual and nocorrection with Intraerror, respectively.

For each patient, the mean value and standard deviation(SD) of RAIF error for different correction action levels were calculated. The population systematic RAIF errors (∑_RAIF_) were calculated from the SD of all the means. The random errors (δ_RAIF_) were calculated from the root mean square (RMS) of all the SDs [21]. The related margins were calculated using the following equation:

$$\text{Margin}=\text{2}.\text{5}\sum_{\text{RAIF}}+0.\text{7}\delta_{\text{RAIF}}$$

which is reported by Van Herk[22]. For the analysis of different CALs, the 3 mmCAL was used as reference and compared with other CALs using t-test.

## Results

Of the ten patients, five had CBCT images for each fraction, and the others five had CBCT images every other fraction due to the limitation of machine availability. A total of 150 CBCT image data sets were collected, with 75 before treatment and 75 after treatment.

### Setup errors from the first CBCT

All initial setup errors based on skin markers were within 10 mm. Table 1 summarizes the systematic, random setup errors and margins for the different correction action levels. The margins were calculated using the same equation as described in the previous section [22]. The systematic and random setup errors for positioning patient with skin markers were 2.1 mm and 1.8 mm in the LR direction, 3.1 mm and 2.3 mm in the SI direction and 2.3 mm and 2.0 mm in the AP direction. Thus, the margins for skin markers setup were 6.5 mm, 9.4 mm and 7.2 mm in the LR, SI and AP directions, respectively.

**Table 1:** The systematic and random setup errors and setup margins in the lateral (LR), superior-inferior (SI) and anterior-posterior (AP) directions with different correction action levels (CALs)(based on the 75 pre-treatment CBCT data sets).

**CAL**	**LR**			**SI**			**AP**		
	**Systematic setup error (mm)**	**Random setup error (mm)**	**Margin (mm)**	**Systematic setup error (mm)**	**Random setup error (mm)**	**Margin (mm)**	**Systematic setup error (mm)**	**Random setup error (mm)**	**Margin (mm)**
	
3 mmCAL	0.9	1.2	3.1	0.9	1.4	3.2	0.7	1.1	2.5
4 mmCAL	1.1	1.5	3.8	1.2	1.7	4.2	0.9	1.2	3.1
5 mmCAL	1.3	1.6	4.4	1.7	1.9	5.6	1.5	1.6	4.9
6 mmCAL	1.6	1.8	5.3	2.1	1.9	6.6	1.9	1.6	5.9
nocorrection	2.1	1.8	6.5	3.1	2.3	9.4	2.3	2.0	7.2

Both the systematic and random setup errors showed a decrease in magnitude with stricter action levels. The maximum systematic errors decreased from 3.1 mm (nocorrection) to 0.9 mm (3 mmCAL); and the maximum random errors decreased from 2.3 mm (nocorrection) to 1.1 mm (3 mmCAL). Compared to the random errors, the systematic errors presented a larger decrease with stricter action levels.

### Errors detected by the post-treatment CBCT images and corresponding margins

For the whole group, the mean and SD for the Intraerror is 1.5 ± 2.6 mm in the LR direction, 0.1 ± 2.6 mm in the SI direction and -0.8 ± 2.6 mm in the AP direction. The RAIF errors at actual 3 mmCAL and hypothetical CALs were then calculated.

**Figure 1:**
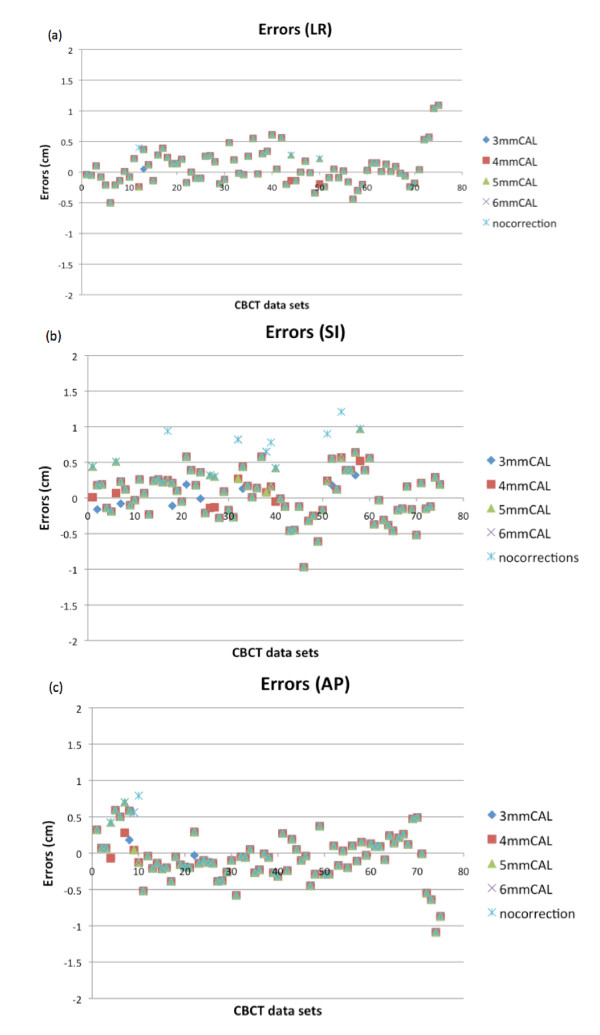
**The distributions of detected and calculated errors based on the 2^nd ^CBCT images for different correction action levels (CALs)**. (a), (b) and (c) show the detected errors in the LR, SI and AP directions, respectively.

Figure 1 shows the distribution of RAIF errors. Most of the RAIF errors (94.8%) were within 7.0 mm in the 3 mmCAL. For the total 75 fractions, at the 3 mmCAL and 4 mmCAL, all RAIF errors were within 10 mm except in 3 (2 in the LR direction and 1 in the AP direction). At 5 mmCAL, 6 mmCAL and nocorrection, the number of fractions with RAIF errors in one direction of 10 mm or above were 3, 4, and 7 respectively.

Table 2 shows the systematic, random RAIF errors and corresponding margins to the different CALs. Similar to the results in the pretreatment CBCT images with skin marker setup, stricter action levels resulted in smaller RAIF errors, except for the LR direction which was not statistically significant (P > 0.05). In the AP direction, the increase in the systematic RAIF error from 3 mmCAL to 4 mmCAL, 5 mmCAL, 6 mmCAL and nocorrection was statistically significant (p = 0.04,0.00, 0.00, 0.00, respectively). In the SI direction, however, statistical difference of the increase of systemic error was only found when the 3 mmCAL was increased to 6 mmCAL and nocorrection (p = 0.026 and 0.021, respectively).

**Table 2:** The systematic, random RAIF errors and corresponding margins in the lateral (LR), superior-inferior (SI) and anterior-posterior (AP) directions with different correction action levels (CALs).

**CAL**	**LR**			**SI**			**AP**		
	**Systematic RAIF error(mm)**	**Random RAIF error(mm)**	**Margin (mm)**	**Systematic RAIF error(mm)**	**Random RAIF error(mm)**	**Margin (mm)**	**Systematic RAIF error(mm)**	**Random RAIF error(mm)**	**Margin (mm)**
	
3 mmCAL	2.5	2.3	7.9	2.0	2.0	6.4	2.3	2.2	7.3
4 mmCAL	2.5	2.5	8.0	2.2	2.3	7.1	2.5	2.5	8.0
5 mmCAL	2.4	2.9	8.0	2.5	2.5	8.0	3.0	2.9	9.5
6 mmCAL	2.3	3.1	7.9	2.9	2.8	9.2	3.3	2.8	10.2
nocorrection	2.3	3.1	7.9	3.3	3.2	10.5	4.3	2.8	12.7

Based on the 3 mmCAL, the CTV-PTV margins with 7.9 mm in the LR, 6.4 mm in the SI and 7.3 mm in the AP direction were required to compensate for the RAIF errors. Maximum CTV-PTV margin was <10 mm in all directions for the 3 mmCAL, 4 mmCAL and 5 mmCAL; 10.2 mm in the AP direction for the 6 mmCAL; 12.7 mm in the AP direction for nocorrection (table 2). For the total 75 CBCT image data sets, the percentage of fractions with shifts smaller than 10 mm in any of the directions were 97.3%, 97.3%, 96%, 94.7%, and 90.7% for 3 mmCAL, 4 mmCAL, 5 mmCAL, 6 mmCAL and nocorrection, respectively.

## Discussion

In this study, we calculated the RAIF errors and corresponding margins with different correction action levels in the CBCT guided APBI. We found that long treatment time and couch rotation in external beam APBI delivery may affect the accuracy of treatment delivery.

APBI using 3-D CRT has demonstrated its superiority in target coverage and dose homogeneity compared with brachytherapy. In order to minimize the unnecessary irradiation to normal tissues, efforts should be made as to reduce the set-up errors and intra-fractional motion, which are two major components in determining the optimal margin. Pretreatment CBCT is helpful in reducing the initial set-up error, while it is not sufficient to determine which margin should be applied as residual error and intra-fractional motion may have their impact on treatment accuracy.

Overall, we found the initial setup margins were 6.5, 9.4 and 7.2 mm in the LR, SI and AP directions, respectively. White et al. reported the systematic setup error based on the skin-marker setup were 2.7, 2.4, and 1.7 mm and random errors were 2.4, 2.9 and 2.2 mm in the LR, SI and AP directions, respectively [20], which made the total setup margins of 8.4 mm, 8.0 mm and 5.8 mm in the LR, SI and AP direction respectively. Both their data and our study have shown that the 10 mm setup margin is feasible for skin-marker setup without considering the residual and intra-fractional motions. Also, they reported that the systematic and random setup errors could be reduced to 0.8 and 1.5 mm in the LR direction, 0.7 and 1.6 mm in the SI direction and 0.8 and 1.5 mm in the AP direction with the 3 mmCAL in the CBCT guidance, respectively. By testing the magnitude of error with different CALs, a correlation of increased error with increasing CALs was found. Further to their study, we wish to find the impact of different CALs on overall residual and intro-fractional errors, which constitute a more reasonable prediction on CTV-PTV margin. We did not start with CALs less than 3 mm as it has been proved that no further reduction of set-up errors could be found when smaller CALs were applied.

Evidently, the implementation of CBCT imaging is important in reducing the initial patient setup errors [20,23]. Margins with 3 mmCAL before treatment were almost half to those with no correction. However, after treatment delivered with 3 mmCAL, the systematic RAIF errors were 2.5, 2.0 and 2.3 mm and the random RAIF errors were 2.3, 2.0 and 2.2 mm in the LR, SI and AP directions, respectively, which were almost similar to the skin-marker setup error detected by pretreatment CBCT images. Such significant changes confirm our hypothesis that the long treatment time and couch rotation can diminish the benefit of pretreatment image guidance. Position verification and correction during the treatment delivery may reduce these errors. The treatment position in our study was both arms raised symmetrically above the patient's head, thus, the chance of LR displacement maybe less than in the AP and SI directions, which were influenced by the respiration and the minor deviation of arm abduction angle, respectively. Therefore, we did not observe a statistical difference of RAIF change with increased CALs in the LR direction.

Based on the formula of Van Herk et al.[22], the margins in the LR, SI and AP directions for the skin marker setup were 6.5,9.4 and 7.2 mm with the data of pretreatment CBCT images, while increased to 7.9,10.5 and 12.7 mm when post-treatment CBCT data were integrated. This finding suggests that the skin marker setup is not sufficient for the safe delivery of APBI if 10-mm CTV to PTV setup margin is used. Instead, a CTV to PTV margin of at least 13 mm is necessary to account for both the initial setup errors and intrafractional errors. A10-mm CTV to PTV margin can be used if the online CBCT guided correction is performed with CALs of 5 mm or smaller for guaranteeing 95% of the fractions have the RAIF errors within 10 mm.

The image registration plays an important role in evaluating the setup errors. Three registration algorithms are implemented in the XVI system: manual, bone and grey. The details of the algorithms have been well described [18,24]. Although the grey algorithm method can achieve a good registration, some fine adjustments are still helpful in most fractions. In this study we combined the automatic grey algorithm method with fine manual adjustment. Baglan et al. demonstrated a strong correlation between the chest wall or rib position and clip position [12]. Weed et al. showed that clips were good radiographic surrogate for the lumpectomy cavity in the image-guided APBI[25]. Topolnjak et al. reported that the uncertainties in the position of the excision cavity could be reduced by using registration of the breast surface[26]. Considering the short treatment duration, we did not study specifically the deformation of breast and surgical cavity between planning CT and CBCTs, and we combined the information of chest wall, clips and skin as the parameters of registration.

One limitation of the current study is that we did not acquire CBCT images after correction; instead we used a method of calculating the result by assuming the 3 mmCAL. The calculated systematic and random setup errors were 0.9 and 1.2 mm in the LR direction, 0.7 and 1.4 mm in the SI direction and 0.7 and 1.1 mm in the AP direction, which had a good agreement to White's residual errors [23]. This confirms the feasibility of using such method for residual errors calculation. Moreover, any residual error with different CALs in one patient can only be tested instead of being measured. The post-treatment CBCT data set had the information of both the residual and intrafraction errors, thus it is reasonable to remove the residual errors to get the intrafractional errors. Another limitation is that we did not integrate the information from rotational errors due to the limitation of the current treatment couch. Although we postulate the actual margins may be less if rotational errors will be corrected, we have no data to confirm that until the result of our further study which will focus on the rotational errors after the installation of 6-degree couch.

Both planning CT and CBCT in our study were acquired in free breathing mode, therefore, the setup errors observed here also accounted for respiratory motion. Baglan et al. showed that a CTV to PTV margin of 10 mm was sufficient for most patients treated with APBI in free breathing [9]. Further to their findings, after we had analyzed in detail the 75 sets of CBCT images, we found that a 10-mm CTV to PTV margin is sufficient for more than 95% of fractions with CAL of 5 mm or less if the residual and intrafractional errors are considered. Actually, the margins would be larger if the potential impacts of breast and tumor bed deformation and delineation errors were involved, but these need further investigation. A CTV to PTV margin of more than 10 mm is required to maintain the desired target coverage for the 6 mmCAL or skin marker setup.

## Conclusion

Residual and intrafractional errors can significantly affect the accuracy of image-guided APBI with nonplanar 3DCRT techniques. The 10-mm margin for skin marker setup was found inadequate for such techniques. A correction action level of 5 mm or less is required to maintain the RAIF errors within 10 mm for more than 95% of fractions. Pre-treatment CBCT guidance is not a guarantee for safe delivery of such treatment despite its known benefits of reducing initial patient setup errors. A patient position verification and correction during the treatment may be a method for the safe treatment delivery Further investigations are ongoing to evaluate the dosimetrical effects of these action levels.

## Competing interests

The authors declare that they have no competing interests.

## Authors' contributions

GC and JYC carried out conception and design, target delineations, image registration, collection and assembly of data, data analysis, manuscript writing. WGH carried out study design, the treatment planning, image registation procedure, data analysis and interpretation, manuscript writing. XLY and ZQP took care of patients. ZZY, XMG, ZMS and GLJ gave advice on the work and participated in study design. All authors read and approved the final manuscript.
